# *In vitro* ruminal fermentation characteristics and utilisable CP supply of sainfoin and birdsfoot trefoil silages and their mixtures with other legumes

**DOI:** 10.1017/S1751731116001816

**Published:** 2016-08-31

**Authors:** A. Grosse Brinkhaus, U. Wyss, Y. Arrigo, M. Girard, G. Bee, J. O. Zeitz, M. Kreuzer, F. Dohme-Meier

**Affiliations:** 1 Agroscope, Institute for Livestock Sciences (ILS), Tioleyre 4, 1725 Posieux, Switzerland; 2 Institute of Agricultural Sciences, ETH Zurich, Universitätstrasse 2, 8092 Zurich, Switzerland

**Keywords:** condensed tannin, legume silage, ruminal fermentation, utilisable CP

## Abstract

The extensive protein degradation occurring during ensiling decreases the nutritive value of silages, but this might be counteracted by tannins. Therefore, silages from two legume species containing condensed tannins (CT) – sainfoin (SF) and birdsfoot trefoil (two cultivars: birdsfoot trefoil, cv. Bull (BTB) and birdsfoot trefoil, cv. Polom) – were compared for their *in vitro* ruminal fermentation characteristics. The effect of combining them with two CT-free legume silages (lucerne (LU) and red clover (RC)) was also determined. The supply of duodenally utilisable CP (uCP) in the forages was emphasised. The legumes were each harvested from three field sites. After 24 h of wilting on the field, the legumes were ensiled in laboratory silos for 86 days. Proximate constituents, silage fermentation characteristics, CT content and CP fractions were determined. Subsequently, silage samples and 1 : 1 mixtures of the CT-containing and CT-free silages were incubated for 24 h in batch cultures using ruminal fluid and buffer (1 : 2, v/v). Each treatment was replicated six times in six runs. The effects on pH, ammonia and volatile fatty acid concentrations, protozoal counts, and total gas and methane production were determined. uCP content was calculated by considering the CP in the silage and the ammonia in the incubation fluid from treatments and blanks. Statistical evaluation compared data from single plants alone and together with that from the mixtures. Among treatments, SF silage contained the least CP and the most CT. The non-protein nitrogen content was lower, favouring neutral detergent soluble and insoluble protein fractions, in the SF and RC silages. Absolute uCP content was lowest in SF and SF mixtures, although the ratio to total CP was the highest. In comparison with LU, the ammonia concentration of the incubation fluid was lower for SF, RC and BTB and for the mixture of SF with LU. The total gas and methane production was similar among the treatments, and the total volatile fatty acid production was decreased with the CT-containing legumes. Protozoal count was increased with the mixtures containing LU and either SF or BTB compared with single LU. In conclusion, compared with the other legumes, SF and RC have similar advantages as they show limited proteolysis during ensiling. In addition, SF supplies more uCP relative to total CP. The CT-containing legumes also differed in their effect on ruminal fermentation and ammonia formation, probably because of their different CT contents. Thus, SF and its mixtures appear promising for improving the protein utilisation of ruminants.

## Implications

Silage has become an important forage type in ruminant nutrition, but proteolysis during ensiling increases the amounts of fast and easily degradable nitrogen (N)-containing compounds in the rumen. This could enhance protein loss, leading to a metabolic and environmental N load. This *in vitro* study revealed that condensed tannins (CT) present in some legume silages may reduce proteolysis during ensiling, thereby lowering the metabolic and environmental load and supplying more utilisable CP (uCP) at the duodenum.

## Introduction

Many European ruminant production systems conserve an increasing proportion of forage as silage because this conservation technique is less weather dependent than haymaking and easily feasible due to modern production technology (Wilkinson and Toivonen, 2003). However, the quality of silage from the same original harvested material can vary widely due to plant respiration, plant enzyme activity, clostridial activity or aerobic microbial activity (Muck, 1988). Protease activity is particularly detrimental as it promotes the formation of soluble N compounds, which could lead to high ammonia (NH_3_) concentrations in ruminants when they are fed (Charmley, 2001). Detoxification of excessive ruminal NH_3_ by ureagenesis is also energy consuming and increases the metabolic load. According to Lobley *et al*. (1995) ureagenesis has been estimated to contribute 13% to 16% to liver energetics in cattle. Wilting of the plant before ensiling can reduce proteolytic processes (Edmunds *et al*., 2014), but the choice of plant may also be important in this respect.

Among legumes, lucerne (LU) is a species that is easily established in dry growing conditions and is characterised by a high yield, whereas red clover (RC) is particularly well adapted for growing conditions with greater humidity (Sheldrick *et al*., 1995). Both of these legumes are rich in CP, but the ruminal degradation rate of the legume CP is generally high. Furthermore, the level of LU CP that is utilisable at the duodenum (uCP; basically metabolisable protein) is further reduced by ensiling (Lüscher *et al*., 2014). This situation might be different in legumes that contain bioactive compounds like CT, which are becoming more important in ruminant nutrition (Waghorn, 2008).

At pH 3.5 to 7, CT form complexes with proteins (Mueller-Harvey, 2006) and carbohydrates (McSweeney *et al*., 2001). These complexes may cause a reduced ruminal degradation rate of nutrients, thereby preventing NH_3_ formation and reducing urinary N loss (Mueller-Harvey, 2006), as well as preserving dietary CP as uCP. In addition, CT bind to the cell coat polymers of some bacteria and can decrease their enzyme activity (Jones *et al*., 1994). Complexes with carbohydrates that result in lower concentrations of H_2_, as well as decreased enzyme activity, might also decrease methane (CH_4_) production (Patra and Saxena, 2011). Another possibility is that CT-containing forages can improve silage quality directly, by decreasing the proteolysis that occurs during ensiling. This is indicated by the lower content of soluble N compounds identified in these silages (Copani *et al*., 2014). Consequently, CT might increase the content of uCP during both the ensiling and ruminal nutrient degradation processes.

A severe disadvantage of the CT-containing legumes growing in temperate climates is their low yield. Therefore, mixtures of high-yield non-CT-containing and medium-yield CT-containing legumes would be particularly attractive as forages. However, it remains unclear whether the positive properties of both types of legumes are conserved, or whether they act synergistically (i.e. the presence of the CT restricts the degradation of the protein from the legumes), antagonistically (i.e. the CT content is too low to be effective) or additively (i.e. the presence of CT restricts the degradation of the protein, but the effect is lower compared with that of the single CT-containing legumes). The lower ruminal NH_3_ concentrations observed when feeding mixtures of LU and sainfoin (SF) (Wang *et al*., 2006) or birdsfoot trefoil (BT) (Williams *et al*., 2010), compared with single LU, suggest a synergistic effect. Nevertheless, to our knowledge, the impact of mixing non-CT-containing and CT-containing legumes on the uCP content has not yet been tested.

Therefore, in the present study, three hypotheses were tested. (i) The silage quality of CT-containing legumes is comparable with that of common non-CT-containing forage legumes. (ii) The CT-containing legumes change ruminal fermentation in a way which increases the amount of uCP. (iii) Mixtures of non-CT-containing legumes with CT-containing legumes act synergistically in this respect. In order to have a wide coverage for testing these hypotheses, two CT-containing species (SF and BT, the latter represented by two cultivars) and two non-CT-containing species (LU and RC) were included in the experiment. Silages of single legumes were prepared at laboratory scale, and ruminal fermentation was simulated in batch cultures using single legumes and 1 : 1 mixtures of CT-containing and non-CT-containing legumes.

## Material and methods

### Silage preparation

LU (*Medicago sativa*, cv. Sanditi), RC (*Trifolium pratense*, cv. Milvus), SF (*Onobrychis viciifolia*, cv. Perly) and two BT cultivars (*Lotus corniculatus*, cv. Bull (BTB) and cv. Polom (BTP)) were sown in April 2012 in Posieux, Switzerland (latitude: 46°46' N, longitude: 07°06' E, altitude: 650 m) in a field of 0.7 ha each. Each field was divided into three plots, and sampling from the plots was done at distances of ~150 m from the next sampling site, in order to obtain three independent batches (replicates) per legume. The sowing density was 26 kg/ha for LU, 23 kg/ha for RC, 160 kg/ha for SF and 30 kg/ha for BTB and BTP each. The germination rate for each legume was as follows: ⩽82% for LU, ⩽85% for RC, ⩽85% for SF, ⩽75% for BTB and ⩽75% for BTP. All legumes were cut at 1300 h at 6 cm above ground on day 73 after seeding. At that time, the SF was at the full flowering stage, whereas LU, RC, BTB and BTP were at the early flowering stage. The weed proportions in the swards were 10%, 14%, 8%, 49% and 49% of harvested biomass for LU, RC, SF, BTB and BTP, respectively. Weeds included *Plantago media* and *Stellaria media*. Sward samples were taken from each legume directly after cutting and after 24 h of wilting. The wilted samples were chopped into 1 to 2 cm pieces with a chaff cutter (Mex GT; Poettinger, Grieskirchen, Austria) and ensiled without additives in silo containers (one 0.5 l, three 1.5 l and one 30 l container/batch and per legume). The silos were covered airtight and stored in a dark room at room temperature. The dry matter (DM) losses were determined by weighing the 1.5 l silos every week on a scale (ID1; Mettler Toledo, Greifensee, Switzerland) and additionally corrected for losses due to evaporation of volatile compounds and respiratory processes according to Weissbach and Kuhla (1995). On day 3, the 0.5 l silos were opened for pH determinations.

After 86 days, the remaining silos were opened. Silage material from the 1.5 l silos was pooled by batch, and two subsamples were taken to determine silage characteristics and protein fractions. The silage material contained in the 30 l silos was subdivided: one part was vacuum packaged and stored at −20°C until used for the *in vitro* incubation. The remaining part, the fresh and wilted material collected before ensiling and one subsample from the 1.5 l silos were lyophilised (Christ, Osterode, Germany) for later analysis of the chemical composition.

### Laboratory analysis of the silages

Silage pH was determined by inserting an electrode (No. 6.0202.110; Metrohm Schweiz AG, Zofingen, Switzerland) connected to an ion metre (pH/ionmeter 692; Metrohm Schweiz AG) into the filtered fluid extracted from 40 g samples shaken for 30 min with 400 ml of distilled water. The NH_3_ content of each extract was also determined with an NH_3_ electrode (No. 6.0506.010; Metrohm Schweiz AG).

Solutions of ~10 g silage, 90 ml distilled water, 2.5 ml Carrez I (18 g K_4_Fe(CN)_6_×3·H_2_O in 500 ml distilled water) and 2.5 ml Carrez II (36 g ZnSO_4_×7·H_2_O in 500 ml distilled water) were shaken and extracted for 3 h to determine the volatile fatty acids (VFA) and short-chain alcohol contents. The acetate, propionate, butyrate, iso-butyrate, valerate, iso-valerate, lactate, propanol, propanediol, butanediol, butanol and ethanol contents of the extracts were analysed by HPLC (Summit1; Dionex, Dublin, Ireland) equipped with a nucleogel ION 300 OA 300×7.8 mm column (Macherey-Nagel GmbH, Düren, Germany) and a Shodex RI-101 refractive index detector (Shodex, Munich, Germany).

The lyophilised samples were milled to pass a 1.0 mm sieve (Brabender mill; Brabender, Duisburg, Germany). The DM was quantified gravimetrically by heating for 15 h at 60°C followed by 3 h at 105°C. The DM values were corrected for losses due to the evaporation of volatile compounds and respiratory processes according to Weissbach and Kuhla (1995). Organic matter (OM) was calculated from the amount of DM and total ash, which was determined by dry-ashing for 4 h at 550°C (Association of Official Analytic Chemists (AOAC), 1995; procedure no. 942.05). An ANKOM 200/220 Fiber Analyzer (ANKOM Technology Cooperation, Fairport, NY, USA) was used for NDF and ADF analysis according to standard protocols (AOAC, 1995; procedure no. 973.18 for ADF and procedure no. 2002.04 for NDF) where NDF was analysed with heat stable amylase and sodium sulphite. Both NDF and ADF were expressed without residual ash (determined by 1 h of incineration). Total N was analysed using the Kjeldahl method (AOAC, 1995; procedure no. 988.05), and CP was calculated as 6.25×N.

The total, soluble, protein-bound and fibre-bound CT contained in the SF, BTB and BTP silages were analysed using the HCl–butanol method described by Terrill *et al*. (1992). In brief, for the first extraction to determine the soluble CT, ~0.5 g ground plant material DM was mixed with an acetone : water solution (7 : 3, v/v) containing ascorbic acid (1 g/l) and diethyl ether. After mixing and centrifugation, the aqueous phase was collected and concentrated. The solid residue containing the insoluble CT fraction was treated with a mix of SDS and 2-mercaptoethanol, heated for 45 min at 95°C and then centrifuged for 15 min at 25 000× **g**. After centrifugation, the supernatant was used to determine the amount of protein-bound CT, and the solid residue was kept to determine the amount of fibre-bound CT. The soluble and protein-bound fractions were heated with a HCl–butanol solution (5 : 95, v/v). The fibre-bound fraction was heated with a HCl–butanol solution (5 : 95, v/v) and SDS-2-mercaptoethanol. After colour development, its intensity was measured with an absorbance spectrometer (Lamda 40; Perkin Elmer Instruments, Waltham, MA, USA) at 550 nm. Standards for concentration of CT were prepared from fresh samples of SF, BTP and BTB by extraction of the CT with an acetone : water solution (7 : 3, v/v) containing ascorbic acid (1 g/l), followed by purification on a Sephadex LH-20 column (Sigma Aldrich, St. Louis, MO, USA). With these extracts, calibration curves were determined. Total phenolic compounds were analysed according to Salminen and Karonen (2011) and were expressed as gallic acid equivalents. The amounts of non-protein N (A), true soluble protein (B_1_), neutral detergent soluble protein (B_2_), protein insoluble in neutral detergent but soluble in acid detergent (B_3_) and protein insoluble in acid detergent (C) were determined using a modified Kjeldahl method, as described by Licitra *et al*. (1996).

### *In vitro* incubation

The Hohenheim Gas Test, developed by Menke and Steingass (1988), was used as an *in vitro* batch culture system. An amount of fresh silage material (single or 1 : 1 mixtures) equivalent to ~200 mg DM of the CT-containing legumes (SF, BTB, BTP) and either LU or RC were incubated separately for each batch in six runs performed on different days. Due to liquid leakage and oxygen entry, the incubation failed for some pistons, which ultimately resulted in an average of five repetitions per batch. Before incubation, the samples were ground to ~5 mm using a Moulinex food processor (Moulinex, Glattpark, Switzerland).

Ruminal fluid was collected before morning feeding from a ruminally cannulated Brown Swiss cow receiving grass hay for *ad libitum* intake. The cow was housed and cared for according to the Swiss legislation for animal welfare (https://www.admin.ch/opc/de/classified-compilation/20080796/index.html). The ruminal fluid was filtered through four layers of gauze and directly mixed with a preheated buffer solution (1 : 2, v/v; Menke and Steingass, 1988), which was flushed with CO_2_ before use. Each modified piston (Soliva and Hess, 2007) was filled with 30 ml of the incubation fluid, closed airtight and incubated for 24 h at 39°C in an incubator with a slow rotation. After 8 h of incubation, the gas volume was read from the calibrated scale printed onto the piston. When it exceeded 50 ml, a 0.150 ml sample was removed through an airtight septum with a sampling injector syringe (Hamilton Company, Reno, NV, USA), and the residual gas was released directly afterwards.

As CH_4_ was not produced linearly during the incubation period, its concentration was directly measured in the gas from the syringes using a gas chromatograph (5890, series II; Hewlett Packard, Avondale, PA, USA) equipped with a 2.34 m×2.3 mm column (mesh size 60/80; Fluka Chemie AG, Buchs, Switzerland) and a flame ionisation detector. After 24 h, the incubation was stopped by removing the pistons from the incubator and the gas volume was recorded. The corrected total gas production was calculated as described by Menke and Steingass (1988) after taking gas produced by blanks and standard samples into account. Total CH_4_ production was calculated with the gas volume and concentrations measured at 8 and 24 h of incubation. It was corrected by the blank samples where the same procedure was applied.

The fluid and the incubation residue were released through the outlet of the piston, and a 10 ml volume of the fluid was collected, mixed with 0.2 ml of 50% H_2_SO_4_ and immediately frozen at −20°C for later analysis of total and individual VFA, as described for the silage samples. In addition, 500 μl of the incubation fluid was mixed with 500 μl of 6% formalin for microscopy determination of the total counts of Holotrich and Entodiniomorph protozoa cells using a Bürker counting chamber (0.1 mm depth; Brand GmbH & Co. KG, Wertheim, Germany). The remaining fluid and the residue were centrifuged together at 20 000×**g** for 30 min at 4°C. The resulting supernatant was used for pH and NH_3_ determination with the appropriate electrodes (pH, HI 223; Hanna Instruments Switzerland AG, Sursee, Switzerland; for NH_3_, No. 6.0506.100, Metrohm Schweiz AG). The NH_3_ electrode was connected to a potentiometer (model 632; Metrohm Schweiz AG).

### Calculations and statistical analysis

The uCP content of the single silages and mixtures was calculated as described by Steingass and Südekum (2013) using the following formula:
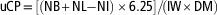
where NB is the average NH_3_–N concentration of the blanks, NL the N content of the silage sample, NI the NH_3_–N concentration of the incubation fluid after 24 h and IW the initial weight of the incubated silage sample. The values of the silage quality and chemical composition were averaged over the three batches per legume, and the means are displayed in the tables. The protein fraction data were subjected to ANOVA using the MIXED procedure of the SAS 9.2 software with treatment as fixed effects. Multiple comparisons among means were calculated using Tukey’s procedure.

Explorative graphics and residual analyses of parametric models led to the decision to apply non-parametric inferential methods for the data obtained from the *in vitro* incubation. The focus of the study was on the treatment comparisons of the single legumes and the mixtures and on the comparison of the single legumes with the respective mixtures; therefore, rank-based ANOVA (Kruskal–Wallis tests) and pairwise comparisons of treatments (Wilcoxon–Mann–Whitney tests) were applied at the significance level of *P*<0.05 using the statistical package of the R 3.1.2 program (R Core Team, 2014). The different batches were treated as replicates for the respective legumes. This resulted, on average, in 15 incubations/legume (*n*=15). The means were trimmed at 10%, and the pooled standard errors of the means are displayed in the tables.

## Results

### Silage characteristics

The pH of all silages declined between the 3^rd^ and 86^th^ days to a pH<5 (Table 1). After 86 days, the pH was lowest in the RC and SF silages, followed by the BTP, BTB and LU silages. Total DM losses were all <50 g/kg and were lowest for the LU silage, followed by the SF, BTB, BTP and RC silages. The DM content was lowest in the RC silage, followed by the BTP, BTB and SF silages, and was highest in the LU silage. The NH_3_ concentration was lowest in the SF silage, followed by the RC, LU, BTP and BTB silages. The lowest concentration of ethanol was found in the RC and LU silages, followed by the BTP, BTB and SF silages. The concentrations of acetate and lactate were lowest for the SF and LU silages, followed by the BTP and BTB silages, and were highest for the RC silage. The other organic acids (propionate, butyrate, iso-butyrate, valerate and iso-valerate) and alcohols (propanol, propanediol, butanediol and butanol) were below the analytical detection limits in all silages (data not shown). The OM content was almost equal across silages. Fibre contents were lowest in the RC silage, but the order in the other silage types differed between NDF (increasing from SF, LU, BTB, BTP) and ADF (increasing from BTB, BTP, LU, SF).

**Table 1:** Characteristics of the legume silages after 86 days of ensiling (*n*=3)

	Legumes				
Items	LU	RC	SF	BTB	BTP
Fermentation variables^1^					
pH^2^_3 days_	5.90	5.01	5.46	5.22	5.19
pH_86 days_	4.84	4.40	4.43	4.62	4.57
DM losses (g/kg)	37.1	47.6	41.2	42.6	42.8
NH_3_	2.96	2.60	1.42	3.12	3.00
Ethanol	1.60	<1.00	3.61	2.96	2.72
Acetate	10.2	28.2	7.17	16.8	16.2
Lactate	50.7	110.3	61.9	68.2	75.5
Composition (g/kg DM)^3^					
DM (wet weight)	490	339	371	361	352
Organic matter	894	884	906	877	891
NDF	436	368	411	438	447
ADF	384	333	403	364	367
Total CP	196	196	139	196	198
Total phenols^4^	12.6	6.74	23.1	13.5	13.0
CT (total)	n.a.	n.a.	136	34.6	22.5
Soluble CT	n.a.	n.a.	37.0	22.3	7.8
Protein-bound CT	n.a.	n.a.	78.7	7.1	11.2
Fibre-bound CT	n.a.	n.a.	20.3	5.2	3.5

LU=lucerne; RC=red clover; SF=sainfoin; BTB=birdsfoot trefoil, cv. Bull; BTP=birdsfoot trefoil, cv. Polom; DM=dry matter; NH_3_=ammonia; CT=condensed tannins; n.a.=not analysed.

1 Analysed in the 1.5 l silos.

2 Analysed in the 0.5 l silos.

3 Analysed in the 30 l silos.

4 Expressed as gallic acid equivalent per kg of DM.

The CP content was by far the lowest in the SF silage (lower, on average, by 28% compared with the other treatments) followed by the RC, LU, BTP and BTB silages. The highest content of total phenolic compounds was found in the SF silage and the lowest in the RC silage, with intermediate levels in the LU, BTP and BTB silages. Among the CT-containing plants, the total CT were lowest in the BTP silage, followed by the BTB silage. The total CT content in the SF silage, was on average, almost five times higher than in the two BT cultivars. Most of the CT in the SF and BTP silages were bound to protein (58% and 50% for SF and BTP, respectively). In the BTB silage, most of the CT were soluble (65%).

The proportion of protein fraction A in the fresh, wilted and ensiled legumes was lowest (*P*<0.05) in SF. When compared with LU, the proportion of fraction A was lower (*P*<0.05) in wilted and ensiled RC and ensiled BTB (Table 2). The proportion of the B_1_ fraction in the fresh legumes was lowest (*P*<0.05) for the SF samples. In the silages, it was lowest for the LU samples, followed by the BTB, RC, BTB and SF samples. Generally, the proportion of the B_2_ fraction showed the greatest decline during the ensiling process and was lowest (*P*<0.05) for the LU, BTB and BTP samples and highest in the SF samples, whereas the RC samples took an intermediate position in the fresh and ensiled samples. The proportion of the B_3_ fraction was consistently lowest (*P*<0.05) in the LU samples and highest in the RC samples, whereas the SF, BTP and BTB samples took intermediate positions during the complete ensiling process. The proportion of the C fraction was highest (*P*<0.05) in the SF samples during the complete ensiling process.

**Table 2:** Protein fractions of the fresh, wilted and ensiled legumes (*n*=3)

	Legumes						
Items	LU	RC	SF	BTB	BTP	SEM	*P*-value
Fresh material							
Total CP (g/kg DM)	196^a^	182^a^	127^b^	187^a^	188^a^	40.9	***
Protein fractions (g/kg of total CP)^1^							
A	190^a^	157^ab^	128^b^	175^ab^	182^a^	10.6	*
B_1_	221^a^	185^ab^	56^c^	159^ab^	135^b^	15.6	***
B_2_	519^b^	550^b^	696^a^	571^b^	585^b^	16.6	***
B_3_	29^b^	63^a^	51^ab^	49^ab^	58^a^	5.9	*
C	41^b^	44^b^	69^a^	46^b^	41^b^	4.9	*
Wilted material							
Total CP (g/kg DM)	178^a^	178^a^	130^b^	179^a^	183^a^	29.4	***
Protein fractions (g/kg of total CP)							
A	306^a^	223^bc^	184^c^	236^abc^	274^ab^	17.5	**
B_1_	57^xy^	61^xy^	35^y^	90^x^	69^xy^	11.4	†
B_2_	510^b^	467^c^	563^a^	472^c^	472^c^	7.6	***
B_3_	71^c^	195^a^	116^bc^	132^b^	133^b^	11.5	***
C	56^b^	53^b^	102^a^	70^ab^	53^b^	8.8	*
Silage^2^							
Total CP (g/kg DM)	183^a^	183^a^	133^b^	187^a^	185^a^	38.2	***
Protein fractions (g/kg of total CP)							
A	589^a^	394^c^	331^d^	536^b^	546^ab^	9.9	***
B_1_	17	20	30	18	29	5.7	Ns
B_2_	292^c^	351^b^	390^a^	308^c^	291^c^	6.5	***
B_3_	44^c^	174^a^	161^a^	80^b^	80^b^	7.1	***
C	59^xy^	60^xy^	89^x^	58^xy^	54^y^	8.2	†

LU=lucerne; RC=red clover; SF=sainfoin; BTB=birdsfoot trefoil, cv. Bull; BTP=birdsfoot trefoil, cv. Polom; DM=dry matter. ^a,b,c,d^Means within a row with different superscripts differ (*P*<0.05). ^x,y^Means within a row with different superscripts tend to differ (*P*<0.10).

1 Protein fractions: A=non-protein N; B_1_=true soluble protein; B_2_=neutral detergent soluble protein; B_3_=protein insoluble in neutral detergent but soluble in acid detergent; C=acid detergent insoluble protein.

2 Analysed in the 1.5 l silos.

†*P*<0.10, *P<0.05, **P<0.01, ***P<0.001, Ns=non-significant (*P*>0.05).

### Differences in the effects on ruminal fermentation among the single legume silages

No difference was noted among the single legume silages in terms of incubation fluid pH or total gas and CH_4_ production, or with ciliate protozoa counts (Table 3). The concentration of total VFA in the incubation fluid was highest (*P*<0.05) for the RC samples and lowest for the SF samples; the other legumes (LU, BTB and BTP) took intermediate positions. The proportion of acetate was highest (*P*<0.05) for the SF samples, intermediate for the LU samples and low for the samples of the three other legumes. The proportion of propionate was highest (*P*<0.05) in the RC samples, lowest in the LU samples and intermediate in the three CT-containing legumes. The proportions of both *n*-butyrate and iso-valerate were lower (*P*<0.05) in the SF samples compared with the other legumes. The proportion of iso-butyrate was highest (*P*<0.05) in the LU and BTB samples, followed by the BTP, RC and SF samples. The *n*-valerate proportion was again highest in the LU samples, but lowest in the SF samples (*P*<0.05), with the other samples falling between. The NH_3_ concentration of the incubation fluid was highest for the LU samples and lowest for the SF samples (*P*<0.05). The three clover legumes took intermediate positions. The calculated content of uCP in the DM was lower (*P*<0.05) in the SF samples when compared with the other legumes. However, the ratio of uCP to total CP was greatest (*P*<0.05) for the SF samples when compared with the other legumes.

**Table 3:** Ruminal fermentation characteristics and utilisable CP at the duodenum when incubating silages from one legume only

	Treatments						
Items	LU	RC	SF	BTB	BTP	SEM	*P*-value
pH	7.57	7.46	7.58	7.43	7.60	0.143	Ns
Total gas (ml/200 mg DM per 24 h)	42.4	43.7	38.4	40.9	39.4	6.22	Ns
CH_4_							
ml/200 mg DM per 24 h	4.68	5.32	5.25	4.94	5.30	0.826	Ns
ml/l total gas	108	119	135	123	131	20.2	Ns
ml/mmol total VFA	66.8	73.8	81.1	75.7	78.3	13.56	Ns
Total protozoa (×10^4^/ml)	1.41	1.86	1.52	1.67	2.02	0.388	Ns
Entodiniomorphs (×10^4^/ml)	1.25	1.60	1.33	1.46	1.79	0.424	Ns
Holotrichs (×10^3^/ml)	0.81	2.22	1.41	1.30	2.22	1.539	Ns
VFA (mmol/l)	70.5^ab^	71.7^a^	64.9^c^	68.1^b^	68.1^b^	2.26	**
Acetate (molar %)	67.9^b^	67.4^c^	69.4^a^	67.3^c^	67.4^c^	0.43	***
Propionate (molar %)	17.8^c^	18.9^a^	18.5^b^	18.5^b^	18.7^ab^	0.22	***
*n*-Butyrate (molar %)	9.59^a^	9.48^a^	8.69^b^	9.69^a^	9.42^a^	0.414	***
Iso-butyrate (molar %)	1.14^a^	1.02^b^	0.86^c^	1.14^a^	1.11^ab^	0.085	***
*n*-Valerate (molar %)	1.56^a^	1.32^c^	1.09^d^	1.48^b^	1.44^b^	0.033	***
Iso-valerate (molar %)	1.88^a^	1.70^a^	1.43^b^	1.87^a^	1.81^a^	0.184	***
NH_3_ (mmol/l)	18.2^a^	16.2^b^	13.0^c^	16.6^b^	16.9^ab^	0.96	***
uCP (g/kg DM)	193^a^	196^a^	140^b^	194^a^	195^a^	4.3	***
uCP : total CP ratio	0.986^b^	0.994^b^	1.013^a^	0.990^b^	0.990^b^	0.0099	***

LU=lucerne; RC=red clover; SF=sainfoin; BTB=birdsfoot trefoil, cv. Bull; BTP=birdsfoot trefoil, cv. Polom; DM=dry matter; CH_4_=methane; VFA=volatile fatty acids; NH_3_=ammonia; uCP=utilisable CP at the duodenum. ^a,b,c,d^Means within a row with different superscripts differ (*P*<0.05). **P<0.01, ***P<0.001, Ns=non-significant (*P*>0.05).

### Effect of mixtures of non-condensed tannins-containing and condensed tannins-containing legume silages compared with the single legume silages

The mixtures of LU (Table 4) and RC (Table 5) with SF, BTB and BTP did not show differences in total gas and CH_4_ production or in pH, total VFA concentration and ciliate protozoa counts in the incubation fluid. The LU–SF mixture, when compared with LU–BTB and LU–BTP mixtures, had a higher proportion of acetate and lower proportions of iso-butyrate, *n*-valerate and iso-valerate, as well as NH_3_ concentration, in the incubation fluid (*P*<0.05). The calculated content of uCP in the DM was lower (*P*<0.05) for the LU–SF mixture compared with the LU–BTB and LU–BTP mixtures, but the ratio of uCP to total CP was higher (*P*<0.05) for the LU–SF compared with the LU–BTP mixture (not significant compared with the LU–BTB mixture). The same effects were observed when comparing mixtures of SF, BTB or BTP and RC, except that the proportions of iso-butyrate, iso-valerate and NH_3_ concentration in the incubation fluid were only numerically lower with the RC–SF mixture, when compared with the RC–BTB and RC–BTP mixtures.

**Table 4:** Effect of mixture (1 : 1) of lucerne and the condensed tannins-containing legumes itself and compared with the single legumes on ruminal fermentation characteristics and supply with utilisable CP at the duodenum

	Treatments										
	LU–SF			LU–BTB			LU–BTP				
Items	Diff. to LU^1^	Observations	Diff. to SF	Diff. to LU	Observations	Diff. to BTB	Diff. to LU	Observations	Diff. to BTP	SEM	*P*-value
pH	−0.17	7.40	−0.18	−0.17^y^	7.40	−0.03	−0.07	7.50	−0.10	0.192	Ns
Total gas (ml/200 mg DM per 24 h)	+0.6	43.0	+4.6	−0.8	41.6	+0.7	−1.3	41.1	+1.7	2.43	Ns
CH_4_											
ml/200 mg DM per 24 h	±0.00	4.68	−0.57	+0.51	5.19	+0.25	+0.20	4.88	−0.42	0.442	Ns
ml/l total gas	+2	110	−25^Z^	+13	121	−2	+12	120	−11	11.0	Ns
ml/mmol total VFA	+2.2	69.0	−12.1	+7.4	74.2	−1.5	+1.3	68.1	−10.2	7.25	Ns
Total protozoa (×10^4^/ml)	+0.35	1.76	+0.24	+0.64^Y^	2.05	+0.38	+0.40	1.81	−0.21	0.270	Ns
Entodiniomorphs (×10^4^/ml)	+0.15	1.40	+0.07	+0.53^Y^	1.78	+0.32	+0.37	1.62	−0.17	0.277	Ns
Holotrichs (×10^3^/ml)	+2.10^Y^	2.91	+1.50	+1.66	2.47	+1.17	+1.07	1.88	−0.34	1.131	Ns
VFA (mmol/l)	−1.4	69.1	+4.2^Z^	+0.7	71.2	+3.1	−1.1	69.4	+1.3	1.94	Ns
Acetate (molar %)	+0.6^Y^	68.5^a^	−0.9^Z^	−0.4	67.5^b^	+0.2	−0.2	67.7^b^	+0.3	0.29	***
Propionate (molar %)	+0.3^Y^	18.1	−0.4^Z^	+0.4^Y^	18.2	−0.3	+0.4^Y^	18.2	−0.5^Z^	0.16	Ns
*n*-Butyrate (molar %)	−0.24	9.35	+0.66^Z^	−0.03	9.56	−0.13	+0.03	9.62	+0.20	0.278	Ns
Iso-butyrate (molar %)	−0.16^Y^	0.98^b^	+0.12^Z^	+0.01	1.15^a^	+0.01	−0.02	1.12^a^	+0.01	0.058	**
*n*-Valerate (molar %)	−0.22^Y^	1.34^b^	+0.25^Z^	−0.02	1.54^a^	+0.06	−0.04	1.52^a^	+0.08^Z^	0.030	***
Iso-valerate (molar %)	−0.27^Y^	1.61^b^	+0.18	−0.03	1.85^a^	−0.02	−0.04	1.84^a^	+0.03	0.131	*
NH_3_ (mmol/l)	−3.6^Y^	14.6^b^	+1.6^Z^	−1.2	17.0^a^	+0.4	−0.7	17.5^a^	+0.6	0.86	***
uCP (g/kg DM)	−25^Y^	168^b^	+28^Z^	+1	194^a^	±0	+1	194^a^	−1	1.8	***
uCP : total CP ratio	+0.011^Y^	0.997^a^	−0.016^Z^	±0.000	0.986^ab^	−0.004	−0.001	0.985^b^	−0.005	0.0072	*

LU=lucerne; SF=sainfoin; BTB=birdsfoot trefoil, cv. Bull; BTP=birdsfoot trefoil, cv. Polom; LU–SF=mixture of LU and SF (1 : 1); LU–BTB=mixture of LU and BTB (1 : 1); LU–BTP=mixture of LU and BTP (1 : 1); DM=dry matter; CH_4_=methane; VFA=volatile fatty acids; NH_3_=ammonia; uCP=utilisable CP at the duodenum. ^a,b^Means within a row with different superscripts differ (*P*<0.05). ^Y,Z^Means of the mixture are different (*P*<0.05) from means found with either LU or SF, BTB and BTP, respectively (data from Table 3).

1 Difference between the value observed in the mixtures and the respective single legumes (Table 3).

*P<0.05, **P<0.01, ***P<0.001, Ns=non-significant (*P*>0.05).

**Table 5:** Effect of mixture (1 : 1) of red clover (RC) and the condensed tannins-containing legumes itself and compared with the single legumes on ruminal fermentation characteristics and supply with utilisable CP at the duodenum

	Treatments										
	RC–SF			RC–BTB			RC–BTP				
Items	Diff. to RC^1^	Observations	Diff. to SF	Diff. to RC	Observations	Diff. to BTB	Diff. to RC	Observation	Diff. to BTP	SEM	*P*-value
pH	−0.17	7.29	−0.29^Z^	−0.16	7.30	−0.13	−0.04	7.42	−0.18^Z^	0.200	Ns
Total gas (ml/200 mg DM per 24 h)	+0.5	44.2	+5.8^Z^	−0.6	43.1	+2.2	−2.5	41.2	+1.8	1.49	Ns
CH_4_											
ml/200 mg DM per 24 h	−0.30	5.02	−0.23	−0.38	4.94	±0.00	−0.38	4.94	−0.36	0.408	Ns
ml/l total gas	−5	114	−21	−3	116	−7	−1	118	−13	9.0	Ns
ml/mmol total VFA	−1.7	72.1	−9.0	−5.1	68.7	−7.0	−4.2	69.6	−8.7	7.42	Ns
Total protozoa (×10^4^/ml)	−0.27	1.59	+0.07	+0.12	1.98	+0.31	−0.03	1.83	−0.19	0.340	Ns
Entodiniomorphs (×10^4^/ml)	−0.16	1.44	+0.11	+0.22	1.82	+0.36	+0.03	1.63	−0.16	0.364	Ns
Holotrichs (×10^3^/ml)	−1.11	1.11	−0.30	−1.41	0.81	−0.49	−0.63	1.59	−0.63	0.996	Ns
VFA (mmol/l)	−1.9	69.8	+4.9^Z^	+0.6	72.3	+4.2^Z^	−0.3	71.4	+3.3	2.36	Ns
Acetate (molar %)	+0.7^Y^	68.1^a^	−1.3^Z^	±0.0	67.4^b^	+0.1	−0.1	67.3^b^	−0.1	0.34	**
Propionate (molar %)	−0.2	18.7	+0.2	±0.0	18.9	+0.4^Z^	−0.1	18.8	+0.1	0.14	Ns
*n*-Butyrate (molar %)	−0.11	9.37	+0.68^Z^	+0.03	9.51	−0.18	+0.14	9.62	+0.20	0.280	Ns
Iso-butyrate (molar %)	−0.05	0.97	+0.11^Z^	+0.02	1.04	−0.10	+0.05	1.07	−0.04	0.070	Ns
*n*-Valerate (molar %)	−0.09^Y^	1.23^b^	+0.14^Z^	+0.06^Y^	1.38^a^	−0.10^Z^	+0.08^Y^	1.40^a^	−0.04^Z^	0.030	***
Iso-valerate (molar %)	−0.09	1.61	+0.18	+0.04	1.74	−0.13	+0.08	1.78	−0.03	0.138	Ns
NH_3_ (mmol/l)	−0.9	15.3	+2.3^Z^	+1.0	17.2	+0.6	+1.4	17.6	+0.7	1.42	Ns
uCP (g/kg DM)	−29^Y^	167^b^	+27^Z^	−3	193^a^	−1	−1	195^a^	±0	2.1	***
uCP : total CP ratio	+0.004	0.998^a^	−0.015^Z^	−0.011	0.983^b^	−0.007	−0.008	0.986^ab^	−0.004	0.0070	*

SF=sainfoin; BTB=birdsfoot trefoil, cv. Bull; BTP=birdsfoot trefoil, cv. Polom; RC–SF=mixture of RC and SF (1 : 1); RC–BTB=mixture of RC and BTB (1 : 1); RC–BTP=mixture of RC and BTP (1 : 1); DM=dry matter; CH_4_=methane; VFA=volatile fatty acids; NH_3_=ammonia; uCP=utilisable CP at the duodenum. ^a,b^Means within a row with different superscripts differ (*P*<0.05). ^Y,Z^Means of the mixture are different (*P*<0.05) from means found with either LU or SF, BTB and BTP, respectively (data from Table 3).

1 Difference between the value observed in the mixtures and the respective single legumes (Table 3).

*P<0.05, **P<0.01, ***P<0.001, Ns=non-significant (*P*>0.05).

Generally, the differences between single silages and the mixtures containing BT silages were much less pronounced than those seen with SF silage mixtures. Furthermore, most of the effects of mixing single silages were additive, and only a few were synergistic or antagonistic compared with the single legumes. In brief, the LU–SF mixture showed higher (*P*<0.05) counts for Holotrich protozoa compared with single LU but not to SF. When compared with LU but not to BTB, LU–BTB mixture had incubation fluid with a lower (*P*<0.05) pH, whereas the counts of total protozoa and of the entodiniomorphid protozoa were higher (*P*<0.05). The RC–SF mixture resulted in a lower (*P*<0.05) pH of the incubation fluid and a higher (*P*<0.05) total gas production compared with single SF but not with RC. Comparisons of RC–BTB with single BTB showed a higher (*P*<0.05) concentration of total VFA in the incubation fluid and comparison of RC–BTP with single BTP showed a lower (*P*<0.05) pH in the incubation fluid. In these traits, no difference occurred when compared with single RC.

## Discussion

### Fermentation quality of the silages

The production of silages with high nutritional value, especially from legumes with high buffering capacity, still appears to be challenging without the use of additives (Muck, 1988). When no fast decline in pH is realised, proteolysis of the plant protein is not suppressed, resulting in high proportions of N as soluble compounds (Muck, 1988). In the present study, the quality of the silages produced in laboratory silos was generally good in all treatments, as evident by the fast decline and the low terminal pH. Consequently, only traces of undesired organic acids, like butyrate, were produced. The DM loss was also negligible, indicating a good compacting and sealing.

### Differences among legumes in their effect on ruminal fermentation and methanogenesis

Although the effect of CT on ruminal carbohydrate degradation is regarded as a secondary anti-nutritional effect compared with that on protein degradation, the formation of complexes of CT with cellulose, hemicellulose and pectin (McSweeney *et al*., 2001) can reduce ruminal fermentation which, in turn, decreases the ruminal VFA and total gas production (Azuhnwi *et al*., 2011). Consistent with this, the VFA concentration was lower in all CT-containing legumes when compared with the non-CT-containing legumes in the present study, and this was most pronounced with SF, which had the highest CT content. Although not statistically significant, the total gas amounts indicate the same.

In cases where the CT decreased the extent of fibre degradation, this could result in a lower CH_4_ production, but a direct anti-methanogenic effect of CT is also possible (Patra and Saxena, 2011). However, although VFA production was lower for the SF samples when compared with the LU and RC samples, the absolute and relative CH_4_ production was not reduced and the change in the VFA profile towards acetate in the SF samples was due to a relative decrease in butyrate. Chung *et al*. (2013) observed no influence of SF on CH_4_ production, and Williams *et al*. (2010) reported no effect for BT. This lack of effect could be due to the CT content, the CT structure, or both, because the content and structure can vary considerably, not only among plant species (Mueller-Harvey, 2006) but also within species, as well as with respect to harvest time and cultivation site (Azuhnwi *et al*., 2013).

Another reason for the lack of an effect on CH_4_ in the SF and BT samples, compared with the LU samples, might be the saponin content of the LU samples. Saponins have the potential to form complexes with sterols in the cell walls of protozoa (Cheeke, 2000) and thereby reduce the CH_4_ production by reducing the number of protozoa or by other mechanisms independent of the presence of protozoa (Hess *et al*., 2003). However, as there was no clear difference in methanogenesis between the LU and RC silages, the specific properties of the LU samples were obviously too weak to have any effect on methanogenesis.

### Differences among legumes in protein degradation during ensiling and ruminal fermentation

Forage diets that are characterised by easily degradable protein, as is the case with many silages, promote the formation of NH_3_ in the rumen. NH_3_ cannot be efficiently used by the ruminal microbes in case of excessive dietary CP or deficient fermentable OM supply or both. The surplus NH_3_ will be absorbed by the ruminant and will need to undergo detoxification into urea in the liver and be excreted in the urine. Excessive NH_3_ absorption therefore could result in metabolic stress in the animal due to its effects on liver health and the costs in terms of energy expenditure (Lobley *et al*., 1995). This is also an undesirable outcome in environmental terms, as the urea in urine is easily transformed into NH_3_, nitrate and nitrous oxides during manure storage and distribution on the field (Dijkstra *et al*., 2013). Wilting of plants before ensiling can be effective in reducing the amount of protein fraction A in favour of the B_2_ and B_3_ protein fractions. This has the effect of counteracting the excessive production of NH_3_ in the rumen and increasing the content of uCP, as ruminal undegradable protein is mainly composed of the B_3_ and C fractions (Edmunds *et al*., 2014).

In the present study, the silages prepared from SF seemed to decelerate proteolysis during the ensiling process. Although the non-protein N fraction A was already lower in fresh SF material, the proportion of fraction A was markedly lower in the SF silage than was observed in the other legume silages. In addition, the protein from fractions B_1_ and B_2_ shifted to the B_3_ and C protein fractions during the ensiling of SF and resulted in the highest ratio of uCP to total CP when compared with the other silages. Copani *et al*. (2014) similarly found a lower content of soluble N in SF silage compared with timothy grass silage, and Scharenberg *et al*. (2007) observed a higher ratio of uCP to total CP in SF silage compared with BT and chicory silage. CT form stable complexes with proteins (Mueller-Harvey, 2006) and thereby decelerate or even prevent ruminal degradation of these proteins, as microbes are unable to cleave these complexes (McSweeney *et al*., 2001). The low NH_3_ concentration and the reduced proportion of iso-VFA in the incubation fluid of SF silage compared with LU indicates an interaction of proteins with CT in the present study. However, the low NH_3_ concentration observed in the SF samples could result from its concomitantly low CP content and proportion of fraction A or its high CT content compared with the other legume silages. Alternatively, it could be a result of a later developmental stage of the plant at harvest or a combination of all these possibilities.

Apart from CT, other bioactive substances are known to reduce proteolysis; for example, the polyphenol oxidase is an enzyme that catalyses the formation of o-quinones (Lee *et al*., 2008). RC contains polyphenol oxidase as shown by Copani *et al*. (2014). These authors also reported a lower amount of soluble N in RC silage (33% of total N) compared with timothy grass silage (55% of total N). The non-protein N fraction A in the RC silage in the present study was lower than that found in the LU silage. The NH_3_ concentration in the incubation fluid from RC was also lower than that found in the fluid from LU, indicating an effect of o-quinones on ruminal protein degradation or silage fermentation or both, resulting in a lower proportion of protein fraction A that in turn reduced the NH_3_ concentration.

The small effect of CT on proteolysis in the BTB and BTP silages compared with SF silage, and the especially low effect of the BTP silages on NH_3_ concentration in the incubation fluid, were most likely the result of too low CT contents, partially caused by the high weed proportions in the swards. However, this effect could partly be a result of different CT structures. CT consist of varying proportions of prodelphinidins and procyanidins, which differ in the size of their polymers. These differences can influence their reactivity to form complexes with other polymers (Patra and Saxena, 2011). Therefore, differences between SF and BT would be expected, as the main CT in SF are prodelphinidins, whereas they are procyanidins in BT (compiled by Mueller-Harvey, 2006). Furthermore, the two BT cultivars differed in their CT fractions. A larger part of the CT in BTB was soluble, whereas about half of the CT in BTP were bound to proteins; this difference might explain the contrasting effectiveness of the two cultivars.

Complexes with CT are assumed to be released below pH 4 (Mangan, 1988), as it is the case in the abomasum. Ideally, therefore, the amount of undegradable protein and hence the amount of uCP (Waghorn, 2008) should be increased so that it contributes to the metabolisable protein supply. The ratio of uCP to total CP was indeed higher for SF than for all other silages tested in the present study, which suggests that this legume silage might improve the dietary protein utilisation and animal performance in diets that are limited in uCP but not in energy.

### Type of response to mixtures in ruminal fermentation compared with effects of single legume silages

Mixing of forage plants may help to improve the forage quality by combining the positive effects of different plants. Ideally, the bioactive compounds present in some plants would act additively or synergistically when mixed and would improve the fermentation characteristics when compared with each single plant (Jayanegara *et al*., 2013). In the present study, no completely synergistic effects (i.e. significantly different from both respective single legumes) were observed. Nevertheless, some incompletely synergistic effects (significantly different from one single legume and numerically different in the same direction from the other single legume) were observed with mixtures of a CT-containing legume with RC or LU. These incompletely synergistic effects were manifested as a greater gas production (RC–SF) and a greater VFA concentration (RC–BTB), whereas the mixtures of LU–SF and LU–BTB showed greater numbers of protozoa. The lack of fully synergistic effects is most likely due to the low CT content of the mixtures, which is consistent with other literature reporting no synergistic effects of mixtures of SF and BT with LU (Williams *et al*., 2010; Chung *et al*., 2013). Nevertheless, synergistic effects may not be the only benefits, as additive effects (where the value of the mixture is in the middle, between the values of the two respective single legumes) could also have a positive impact on forage quality. For instance, the mixtures of SF with LU and RC, when compared with single LU and RC, reduced protein degradation to NH_3_ and enhanced the content of uCP.

## Conclusion

The present findings showed that, by wilting the material before ensiling, it is possible to produce silages of the same quality as with common non-CT-containing legumes, even though no additives were applied in any of the silages. This confirms the first hypothesis. SF (and RC) silages resulted in a more favourable protein fraction profile because ensiling these plants proportionately decreased non-protein N and increased B_3_ protein when compared with LU silage. This could have resulted from the effect of bioactive compounds (CT and polyphenol oxidase). In the case of SF, this translated into a higher proportionate supply of uCP at the duodenum. Compared with SF, the BT cultivars, which contained much less CT, had little effect on protein-related traits. This shows that with SF, the second hypothesis was correct, but that it cannot be generalised to all CT-containing legumes and to low CT levels. Some positive effects were obtained by mixing CT-containing and non-CT-containing legumes in terms of lowering the ruminal NH_3_ concentration and increasing the supply of uCP, which could motivate farmers to integrate CT-containing legumes into ruminant diets. These findings confirm the third hypothesis. Mixing CT-containing and non-CT-containing legumes already during the ensiling process might reduce N losses even more and improve forage quality further. This has to be clarified by further research.
